# Apparent regional differences in the spectrum of *BARD1* pathogenic variants in Spanish population and importance of copy number variants

**DOI:** 10.1038/s41598-022-12480-2

**Published:** 2022-05-20

**Authors:** B. Benito-Sánchez, A. Barroso, V. Fernández, F. Mercadillo, R. Núñez-Torres, G. Pita, L. Pombo, R. Morales-Chamorro, J. M. Cano-Cano, M. Urioste, A. González-Neira, A. Osorio

**Affiliations:** 1 Familial Cancer Clinical Unit, Human Cancer Genetics Programme, Spanish National Cancer Research Centre (CNIO), 28029 Madrid, Spain; 2 Human Genotyping Unit (CEGEN), Human Cancer Genetics Programme, Spanish National Cancer Research Centre (CNIO), 28029 Madrid, Spain; 3 Medical Oncology Section, Universitary Hospital Complex of Albacete, Albacete, Spain; 4 Medical Oncology Section, Hospitalary Compex La Mancha Centro, Alcázar de San Juan, Ciudad Real, Spain; 5 Medical Oncology Service, Universitary General Hospital of Ciudad Real, Ciudad Real, Spain; 6 Spanish Network On Rare Diseases (CIBERER), 28029 Madrid, Spain; 7 Familial Cancer Clinical Unit, Human Cancer Genetics Programme, Spanish National Cancer Research Centre (CNIO), C/Melchor Fernández Almagro 3, 29029 Madrid, Spain

**Keywords:** Cancer genetics, Breast cancer

## Abstract

Only up to 25% of the cases in which there is a familial aggregation of breast and/or ovarian cancer are explained by germline mutations in the well-known *BRCA1* and *BRCA2* high-risk genes. Recently, the BRCA1-associated ring domain (*BARD1*), that partners *BRCA1* in DNA repair, has been confirmed as a moderate-risk breast cancer susceptibility gene. Taking advantage of next-generation sequencing techniques, and with the purpose of defining the whole spectrum of possible pathogenic variants (PVs) in this gene, here we have performed a comprehensive mutational analysis of *BARD1* in a cohort of 1946 Spanish patients who fulfilled criteria to be tested for germline pathogenic mutations in *BRCA1* and *BRCA2*. We identified 22 different rare germline variants, being 5 of them clearly pathogenic or likely pathogenic large deletions, which account for 0.26% of the patients tested. Our results show that the prevalence and spectrum of mutations in the *BARD1* gene might vary between different regions of Spain and expose the relevance to test for copy number variations.

## Introduction

Breast cancer (BC) is the most common malignancy in women and the second cause of death due to cancer in this sex in the world^1^. *BRCA1* and *BRCA2* are the main high-risk susceptibility genes responsible for Hereditary Breast and Ovarian Cancer (HBOC), however their germline mutations only explain up to 25% of the cases in which a familial aggregation of these diseases is observed. Other moderate-high susceptibility genes have been identified in recent years, whose association with breast and/or ovarian cancer susceptiblity has been accurately established, thanks to advances in next-generation sequencing (NGS) technologies^2,3^. Recently, the two largest BC case–control studies published so far, have confirmed those genes that have an actual association with the disease, and therefore should be included in diagnostic testing, in addition to *BRCA1/2*. These genes are *ATM, BARD1, CDH1, CHEK2, PALB2, RAD51C, RAD51D* and *TP53*^4,5^. All of those were already considered clinically relevant^6–8^, except *BARD1*, whose role in BC susceptibility had not been so clearly established until now.

The BRCA1-associated ring domain (*BARD1*) gene encodes a 777 amino acid protein which was discovered in 1996 to directly interact with BRCA1, through their homologous N-terminal RING domains, to form a stable complex^9^. BARD1 also contains two tandem conserved C-terminal BRCT domains shared with BRCA1, and four tandem ankyrin repeats. The tumour suppressor complex BRCA1-BARD1 interacts with DNA and other DNA damage response factors in order to repair double-strand breaks, including damaged replication forks, via homologous recombination. Besides, the heterodimer acts as an efficient E3 ubiquitin ligase, targeting proteins involved in cell-cycle, genome stability and hormone signaling^10–13^. BARD1 has several BRCA1-independent functions, including the inhibition of mRNA maturation and the induction of apoptosis by means of p53 stabilization^14^. Furthermore, *BARD1* spliced isoforms lacking regions of functional domains, have been found overexpressed in some breast and ovarian tumors in comparison with the full-length protein, and have shown oncogenic properties^15^.

The two studies by Dorling et al. and Hu et al. have definitely confirmed the role of *BARD1* as a moderate BC susceptibility gene (OR: 2.09; 95% CI, 1.35–3.23; *P* = 0.00098, for protein-truncation variants^4^). These studies also report a stronger association of pathogenic variants (PVs) in *BARD1* with ER-negative tumors and, more remarkably, with triple-negative breast cancer (TNBC).

Numerous studies have been performed in the last years aiming to define the prevalence of mutations in the *BARD1* gene in different cohorts^16^. However, to our knowledge, none of them has taken in account the whole spectrum of possible pathogenic variants (PV) in the gene. Regarding the Spanish population, only one study has investigated the prevalence of *BARD1* PVs in an important number of patients, focusing on the region of Catalonia^17^. With the purpose of continuing to deepen our knowledge of the gene in breast and ovarian cancer predisposition, here we report a comprehensive mutational analysis of the *BARD1* gene in a cohort of 1946 patients who fulfilled criteria to be tested for pathogenic mutations in the *BRCA1/2* genes^18^ from different regions of Spain.

## Results

**Figure 1:**
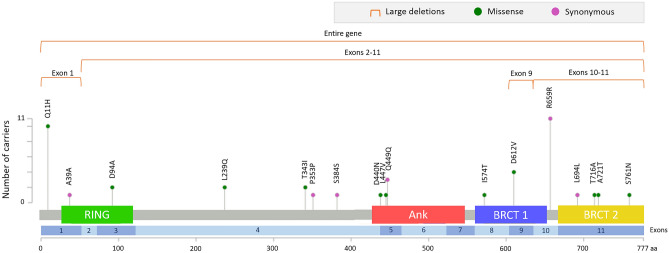
Map of the BARD1 protein domains showing the location of rare germline variants identified in our cohort. The graphic was generated with MutationMapper tool at cBioPortal for Cancer Genomics (https://cbioportal.org/mutation_mapper).

Among the 1946 patients screened using targeted NGS, we identified 22 different *BARD1* rare germline variants, 5 Copy Number Variants (CNV), 11 missenses and 6 synonymous, distributed in 49 patients (5 carried a CNV, 26 a missense and 18 a synonymous variant). Strickingly, we did not find any frameshift, nonsense nor splicing variants. The position across the protein of the detected variants is represented in Fig. 1. Each patient carried one single rare variant, except for one case that presented the variants c.1339C > G; p.Leu447Val and c.1059C > G; p.Pro353 simultaneously. c.1977A > G; p.Arg659 = was found in 11 patients, representing the most recurrent variant in our population of study; and c.33G > T; p.Gln11His was the second most common appearing in 10 cases. When applying a Fisher exact test comparing the number of individuals harbouring each of these two variants in our series of cases vs. local controls, no statistically significant differences were found (*p* = 0.318 and 0.492 respectively), suggesting that these variants are not potentially associated with BC risk.

All variants found were classified according to their pathogenicity based on ACMG guidelines and taking into consideration ClinVar database (https://ncbi.nlm.nih.gov/clinvar/), literature search and Franklin Genoox platform (https://franklin.genoox.com/clinical-db/home) variant interpretation.

### 0.26% of our cohort of Spanish patients fulfilling criteria for *BRCA1/2* testing present pathogenic/likely pathogenic variants in the *BARD1* gene

Only the five CNVs detected in our series were considered as pathogenic or likely pathogenic following ACMG guidelines. CNVs were detected from NGS data, based on the depth of coverage which is correlated with the copy number of the region^19^. All of them were predicted to cause an out of frame RNA, predicted to derive in an incorrect translation of aminoacids that ends up in the appearance of a premature stop codon, involving the loss of an important functional domain of the protein (Fig. 1 and Supplementary Fig. S1). Each one of the five large deletions reported was present just once in our cohort, giving a prevalence of 0.00257 (5/1946), and were not present in control samples. For establishing PM2 (Pathogenic Moderate 2) criteria, (absent from controls in Exome Sequencing Project, 1000 Genomes or ExAC) we took in account structural variants (SVs) detected in *BARD1* over more than 21,000 alleles from gnomAD SVs v2.1 dataset. We also took in account data from Fabulous Ladies Over Seventy (FLOSSIES, https://whi.color.com/) database, from which along 9884 noncancer controls, the only deletion reported (once) comprised exons 4–11 with an overall frequency of 0.0001012 in contrast to our 0.00257 prevalence of large deletions in the cases studied. As local controls, we included 1119 DNA samples from Spanish individuals provided by Spanish National Biobank (BNADN), that had been genotyped using Illumina Global Screening Array v1.0 + MD containing 700.078 SNPs. We made use of these data to analyse CNVs in the *BARD1* gene, and no CNVs were detected.

Of note, out of the five CNVs detected, only deletion of exon 1 and deletion of the entire gene have been previously described^20–22^. In this study, we report for the first time the deletion of exons 10–11, deletion of exon 9 and deletion of exons 2–11.

Carriers of these PVs were women diagnosed with BC, whose genotype and phenotype data are summarized in Table 1. A breakdown of the patients based on the risk criterion fulfilled, and percentage of *BARD1* PVs found in each group are shown in Table 2.

**Table 1:** Genotype and phenotype data of 5 BC patients carrying a large deletion in the *BARD1* gene.

Deletion	ACMG classification^a^	Tumor phenotype	Age at diagnosis	Family history of cancer (Age at diagnosis)	Additional PVs
Entire gene	Pathogenic (PVS1_Stand-alone)	Luminal B, HER2-	35	–	–
Exon 1	Pathogenic (PVS1 + PM2 + PP4)	Luminal A, ER +	68	Mother, BC (68) Maternal female cousin, BC (60)	–
Exons 2–11	Pathogenic (PVS1_Stand alone)	TNBC	54	Mother, CRC (74) Maternal aunt, SC (62)	–
Exon 9	Likely Pathogenic (PVS1_Strong + PM2 + PP4)	Luminal A, ER + PR + Bilateral: luminal A, ER +	48 54	Sister, BC (51) Maternal aunt, BC (88) Paternal uncle, CRC (?) Paternal aunt, BC (50) Paternal aunt, BC (60)	–
Exons 10–11	Pathogenic (PVS1 + PM2 + PP4)	HER2 +	41	Mother, CRC (70) Father, GC (80) Maternal aunt, PC (83) Maternal aunt, GC (70) Maternal female cousin, TNBC (56) Maternal female cousin, CRC (45) Maternal male cousin, GBM (55)	*BRCA2* c.4965C > A; p.Tyr1655X

*PVS1* Pathogenic very strong 1, *PM2* Pathogenic moderate 2, *PP4* Pathogenic supporting 4, *BC* Breast cancer, *CRC* Colorrectal cancer, *SC* Stomach cancer, *GC* Gastric cancer, *PC* Pancreatic cancer, *GBM* Glioblastoma multiforme.

^a^Large deletions were classified following ACMG guidelines adapted for single-gene CNVs^36^.

**Table 2:** Breakdown of the patients included in the study, based on the criteria fulfilled to be screened for pathogenic mutations in *BRCA1* and *BRCA2*.

Phenotype	Number of patients	*BARD1* pathogenic mutations carriers
i) one BC diagnosed at < 40 years	194	1 (0.51%)^d^
ii) ≥ 2 first-degree relatives diagnosed with BC at least one of them at < 50 years	622	1 (0.16%)
iii) one TNBC diagnosed at < 60 years^a^	116	1 (0.86%)
iv) ≥ 3 first-degree BC relatives	233	2 (0.85%)
v) ≥ 1 male BC case^b^	39	–
vi) ≥ 1 ovarian cancer case^c^	447	–
vii) BC not fulfilling i-vi criteria but being bilateral or having any antecedent of pancreatic or prostate cancer	295	–
Total	1946	5 (0.26%)

*BC* Breast cancer, *TNBC* Triple negative breast cancer.

^a^includes only patients without familial antecedents of BC or ovarian cancer, TNBC cases fulfilling any of the other criteria (i-vi), were included in the corresponding group, the total number of TNBC in the whole series is 243.

^b^All male breast cancer cases were included in this group, even if they fulfilled any of the other criteria.

^c^All families containing one ovarian cancer case were included in this group, even if they fulfilled any of the other criteria, except those in which there was a case of male breast cancer that were included in group v.

^d^Number and percentage of patients carrying pathogenic *BARD1* mutations in each phenotypic group.

### Reclassification of rare *BARD1* synonymous variants

Most of the synonymous variants detected in our population were previously described with conflicting interpretations of pathogenicity in ClinVar (Supplementary Table 1). In an effort to add more evidence to the current classification, we carried out a cDNA assay to elucidate their individual effect in splicing. With RNA derived from blood samples of carriers we performed a RT-PCR, followed by an amplification with specific primer pairs, ending up with Sanger sequencing. All the analyzed variants showed biallelic expression at the cDNA level (Supplementary Fig. S2), confirming that none of them caused an alteration in the splicing process, which agrees with benign strong 3 (BS3) criteria from ACMG guidelines. By applying the BS3 evidence we provide an updated classification to benign for all of them.

It is worth mentioning that the c.1977A > G silent change had been previously described to alter several exonic splicing enhancer (ESE) motifs causing the deletion of exons 2 to 9 and resulting in a frame-shift which created a premature stop codon (p.Cys53_Trp635delinsfsX12)^23^. On the contrary, for the only RNA that we had available, out of all the patients carrying this variant, we did not see this alteration (Supplementary Fig. S2). Moreover, this synonymous change resulted to be the rare variant appearing more times in our population of study, which in addition to the global allele frequency shown in GnomAD, support its neutrality (Supplementary Table 1).

### Analysis of rare *BARD1* missense variants

To our knowledge, this is the first study in which a comprehensive reporting of *BARD1* missense variants is performed. Although we did not conduct functional assays, we performed an exhaustive review of the literature to try to add any piece of information that could contribute to their classification (Table 3).

**Table 3:** Summary of rare *BARD1* missense variants identified in the 1946 patients.

DNA level	Protein effect	Our cohort frequency	gnomAD frequency^a^	CSVS frequency^b^	ClinVar^c^	Franklin^d^	Final^e^
c.33G > T	p.Gln11His	0.00513874	0.001634	0.00383693	Benign/Likely benign	Benign	Benign
c.281A > C	p.Asp94Ala	0.00102774	0.00003185	Not present	VUS	VUS	VUS
c.716 T > A	p.Leu239Gln	0.00102774	0.00009244	0.00047801	Likely benign/VUS	VUS	VUS
c.1028C > T	p.Thr343Ile	0.00102774	0.0001168	0.00191479	Benign/VUS	VUS	VUS
c.1318G > A	p.Asp440Asn	0.00051387	0.00000398	Not present	VUS	VUS	VUS
c.1339C > G	p.Leu447Val	0.00051387	0.00007561	Not present	VUS	VUS	VUS
c.1718 T > C	p.Ile573Thr	0.00051387	0.00002476	Not present	VUS	VUS	VUS
c.1835A > T	p.Asp612Val	0.00205549	0.00007434	0.00191479	Likely benign/VUS	VUS	VUS
c.2146A > G	p.Thr716Ala	0.00051387	0.00000795	Not present	VUS	VUS	VUS
c.2161G > A	p.Ala721Thr	0.00051387	0.00002830	Not present	VUS	VUS	VUS
c.2282G > A	p.Ser761Asn	0.00051387	0.001713	0.00047801	Benign/Likely benign/VUS	Benign	VUS

*VUS* Variant of uncertain significance.

^a^Total allele frequency for all populations described in gnomAD (https://gnomad.broadinstitute.org/).

^b^CSVS = Collaborative Spanish Variant Server. Here we show the frequency of the variants found in a local series of 2094 Spanish unrelated individuals that can be considered as controls.

^c^Aggregate clinical significance from all records at ClinVar (https://ncbi.nlm.nih.gov/clinvar/).

^d^Clinical classification using ACMG criteria generated by the advanced artificial intelligence Franklin by Genoox (https://franklin.genoox.com/clinical-db/home).

^e^Final classification with our own criteria based on previous information and following ACGM guidelines.

p.Thr716Ala is classified as benign following ACMG guidelines, relying on its relatively high frequency in control populations extracted from independent databases. In our cohort, p.Thr716Ala was only present in one patient. Moreover, several published studies have designated p.Thr716Ala as deleterious, arguing that it abolishes binding to OLA1, a protein interacting in BRCA1-BARD1 complex, leading to the alteration of BARD1 tumor suppressor activity^24–26^. Taking this in account, we consider that it should be regarded as VUS.

A total of 105 *BARD1* missense and truncation variants have been previously tested in a functional assay for homology-directed repair (HDR) alteration^27,28^. Of our detected missense variants, p.Asp612Val, p.Thr343Ile and p.Leu239Gln were described as functional, although for these two last variants a significantly increased loss of heterozygosity (LOH) has been reported. In addition, they recognized p.Thr716Ala as functional, while other studies had described this variant as deleterious as mentioned before. This suggests that HDR assay, although informative, might not be sufficient for classifying *BARD1* variants.

## Discussion

Familial BC is a polygenic disease that can be partially explained, by the effect of PVs distributed over several susceptibility genes with different levels of risk^29^. In this context, *BARD1* has been recently confirmed as a moderate-risk BC gene by two large case–control studies, sorting out the uncertainity about its association with the disease^4,5^. With the aim of establishing the mutational spectrum of *BARD1* in the Spanish population in order to improve genetic diagnosis, we screened the gene for PVs by a multigene panel analysis in a series of 1946 Spanish patients who fulfilled criteria to be tested for germline mutations in the *BRCA1/2* genes. To our knowledge, this is the first study that comprehensively reports all types of rare variants in the *BARD1* gene, subsequently discovering 5 large deletions, 11 missense and 6 synonymous from which only the large deletions were finally regarded as pathogenic or likely pathogenic. Surprisingly, no frameshift, nonsense nor splicing variants were found, which could be due to the limited size of our cohort or to populations or even regional differences in the spectrum of *BARD1* mutations.

Previously, another study investigated the presence of germline PVs within the *BARD1* gene in the Spanish population^17^. Among 4015 patients with clinical suspicion of HBOC, they identified 19 PVs: 8 truncating, 1 splicing and 2 large deletion, ending up with a PV prevalence of 0.47% which approximately doubles the 0.22% reported by Couch et al. and the 0.25% described by Suszynska and Kozlowski^16,30^, and triples the 0.13% of protein-truncating variants reported by Dorling et al. and the 0.15% identified by Hu et al.^4,5^. In our cohort, we found a prevalence of 0.26%, which is similar to previous studies, but lower than that reported in the other Spanish cohort. This could be explained by the lower number of patients studied here, or differences in the ascertainment of the cases. These differences in the frequency of mutations, could also be explained by the possibility of variation in the mutational spectrum of *BARD1* between different geographical areas of Spain, as most of our patients proceeded from Madrid and Castilla la Mancha regions, while those reported in Rofes et al. proceeded mainly from Catalonia. These regional differences have been already observed for mutations in the *BRCA1/2* genes, and detecting them is very relevant to improve genetic diagnosis^31^, however, larger studies performed in Spanish population would be necessary to confirm this hypothesis.

Germline CNVs are recognized as the genetic cause of diverse hereditary pathologies, even though their detection via NGS is still challenging^32^. Only a few studies have characterized the CNV landscape within the *BARD1* gene, having reported a total of 8 different large deletions so far in BC or OC patients^17,20–22,33–35^. In our study, we have detected the deletion of exon 1 and the deletion of the entire gene previously reported^20,21^ and, what is more, we have identified for the first time exons 2–11, exon 9 and exons 10–11 deletions present in three BC cases. It is very important to highlight that any deletion can not be assumed to be pathogenic, and special caution should be taken with the ones that affect one exon and/or do not produce a frameshift. In our case, all the large deletions were predicted to cause an out of frame RNA, and classified as pathogenic or likely pathogenic by following the ACMG guidelines adaptation for single-gene copy number variants^36^.

In addition to clearly PVs, to our knowledge, this is the first study in which a comprehensive analysis of all missense and synonymous rare variants is performed. There are already several functional studies reporting that some of these rare variants could be pathogenic^24,25,27,28^. Unfortunately, we were not able to classify most of the *BARD1* missense variants we identified, but we think it is relevant to include them in further screenings and perfom functional assays, as otherwise, the percentage of PVs could be underestimated. Regarding synonymous variants, it is important to carefully analyze the effect of apparently silent changes, taking into consideration that this type of variants are usually classified as (likely) benign, obviating their possible effect in splicing, which could turn out in a missclassification. For all the rare synonymous variants in our population, damaging effect in splicing was experimentally ruled out, which corresponds with BS3 criteria of ACMG guidelines that allowed us to classify them all as definitively benign. These includes the c.1977A > G variant, that had been previously suggested to be deleterious by altering splicing and causing the partial deletion of exons 2–9 of the gene^23^. In our hands, no alteration in splicing was seen for this variant (Supplementary Fig. S2). Due to this discrepancy, these findings should be confirmed. However, the fact that c.1977A > G was present in 11 patients from our cohort, together with a 0.002 global allele frequency described in GnomAD (including one homozygote individual) and an even higher frequency of 0.005 in local controls (Table 3), support our finding in favour of its neutrality. Actually, frequency in controls, exceed the expected for disorder, that is 0.001 for *BARD1* (according to the calculations made by the Franklin Genoox platform). Taking this in account, the variant would meet the BS1 and BP7 criteria and, even in the absence of BS3, could be classified as likely benign.

Finally, many independent studies have associated germline PVs in *BARD1* with high risk of TNBC compared to other BC subtypes^4,5,17,37–39^. This assumption turns out to be of great importance, given that these patients often show a more aggressive disease course, with higher rates of metastases, earlier age at diagnosis and recurrence, and worse survival rates^40^. In our study, only one out of the 5 BC patients who carried a PV, was diagnosed with TNBC, representing 0.4% of the 243 patients that were diagnosed with TNBC in our series, regardless of fulfilling any other criteria. While doubling the overall rate, the reduced size of the TNBC subset does not allow us to drive any conclusion in this regard.

In summary, here we report the second largest study on *BARD1* performed in Spanish population, and the first one in which all possible rare variants are comprehensively reported. Our results show that the prevalence and spectrum of mutations might vary between different regions in Spain, and highlights the large deletions in *BARD1* as important contributors to BC susceptibility in our population. Larger studies and incorporation of independent functional assays are needed in order to properly classify each variant by its clinical impact, which is essential to determine the degree of susceptibility to the disease for each carrier.

## Methods

### Patients

A total of 1946 patients who fulfilled criteria to be tested for germline mutations in the *BRCA1/2* genes were recruited from the Spanish National Cancer Center (CNIO), Hospital General La Mancha Centro, Complejo Hospitalario Universitario de Albacete and Hospital General Universitario de Ciudad Real. Families fulfilled one or more of the following criteria: (i) one BC diagnosed at < 40 years; (ii) ≥ 2 first-degree relatives diagnosed with BC at least one of them at < 50 years; (iii) one TNBC diagnosed at < 60 years; (iv) ≥ 3 first-degree BC relatives; (v) ≥ 1 male BC case; (vi) ≥ 1 ovarian cancer case; (vii) BC not fulfilling i-vi criteria but being bilateral or having any antecedent of pancreatic or prostate cancer (Table 2). Patients included in the study signed an appropriate informed constent and the proposal was approved by the ethics committee at the Fuenlabrada University Hospital, Madrid, Spain. All methods were carried out in accordance with relevant guidelines and regulations.

### Controls

For frequency comparison of single nucleotide change variants detected in our patients, we used data from 125,748 exome sequences and 15,708 whole-genome sequences from unrelated individuals from gnomAD (https://gnomad.broadinstitute.org/) and 2094 Spanish unrelated individuals from the Collaborative Spanish Variant Server (http://csvs.clinbioinfosspa.es/).

For CNV classification, we used data from more than 21,000 alleles from gnomAD SVs v2.1 dataset, 9884 noncancer controls from Fabulous Ladies Over Seventy (FLOSSIES, https://whi.color.com/). We also included a total of 1119 DNA samples from Spanish individuals provided by Spanish National Biobank (BNADN). DNA samples were genotyped using Illumina Global Screening Array v1.0 + MD containing 700.078 SNPs according to the manufacturer's protocols and scanned using a iScan system. GenomeStudio software v2.0.4 (Illumina) was used for genotype calling. Before CNV analysis, *BARD1* region was carefully inspected to ensure enough probe coverage of the gene, resulting in 59 probes thorugh all the gene. CNVs were detected and called using PennCNV v1.0.5 software from genotype, Log R Ratio (LRR) and B Allele Frequency (BAF) data values^41^. *BARD1* gene locus (Chr 2: 215,590,370–215,674,428 GRCh37) and an additional 100 KB up and downstream, were inspected to identify CNVs in this region.

### DNA isolation

Genomic DNA was extracted from peripheral blood samples using Maxwell RSC automated instrument (Promega) according to the manufacturer’s protocol. Purified DNA was quantitated making use of Quant-iT PicoGreen dsDNA reagent (Invitrogen).

### Next-generation sequencing (NGS)

*BARD1* was analyzed within a larger NGS panel, Onco-GeneSGKit (Sistemas Genómicos). Libraries were prepared employing capture-based target enrichment covering 111 genes involved in familial cancer (*ACD, AIP, APC, ATM, ATR, AXIN2, BAP1, BARD1, BLM, BMPR1A, BRCA1, BRCA2, BRIP1, BUB1, CDH1, CDK4, CDKN1B, CDKN2A, CHEK2, DDB2, DICER1, DIS3L2, DKC1, ELANE, EPAS1, EPCAM, ERCC1, ERCC2, ERCC3, ERCC4, ERCC5, ERCC6, FANCA, FANCB, FANCC, FANCD2, FANCE, FANCF, FANCG, FANCI, FANCL, FANCM, FH, FLCN, G6PC3, GFI1, GREM1, HOXB13, JAGN1, KIF1B, KIT, MAX, MDH2, MEN1, MET, MITF, MLH1, MNX1, MSH2, MSH6, MSR1, MUTYH, NBN, NF2, NFIX, NHP2, NOP10, NSD1, NTHL1, PALB2, PARN, PDGFRA, PMS1, POLD1, POLE, POLH, POT1, PRKAR1A, PTCH1, PTEN, RAD50, RAD51, RAD51C, RAD51D, RB1, RECQL, RECQL4, RET, RTEL1, SCG5, SLX4, SMAD4, SMARCA4, STK11, SUFU, TERC, TERT, TINF2, TMEM127, TP53, TSC1, TSC2, UBE2T, VHL, VPS45, WAS, WRN, WT1, XPA, XPC, XRCC2*). Briefly, 50 ng of total gDNA from cases was tagmented and amplified. Quality and quantity assessment of the resulting libraries was performed on a 2100 Bioanalyzer system (Agilent). Libraries were hybridized with capture probes to targeted regions, followed by hybrid capture with streptavidin-coated magnetic beads. Finally, libraries were indexed by PCR and combined in a single run before sequencing on a MiSeq platform (Illumina). The average read depth per sample was 200X, and, on average, 99.9% of the tardeted regions had a coverage of at least 20X.

### Variant calling and classification

FastQ files generated were uploaded in GeneSystems platform (Sistemas Genómicos) to perform variant annotation. GenBank reference sequence NM_000465.3, with numbering starting at the A of the first ATG, was used for *BARD1* variant nomenclature following the Human Genome Variation Society (HGVS) guidelines (http://varnomen.hgvs.org/). All coding exons and at least + /− 25 intronic nucleotides flanking each exon were analysed. Variants showing a minor allele frequency (MAF) < 0.01 in every subpopulation of the Genome Aggregation Database v2.1.1 (gnomAD, https://gnomad.broadinstitute.org/) were considered as “rare” and selected for further analysis.

Sequence variants identified in this work were grouped by their clinical significance according to the American College of Medical Genetics and Genomics (ACMG) standards and guidelines as pathogenic, likely pathogenic, uncertain significance (VUS), benign, or likely benign^42^. In the case of large deletions, the adaptation of this classification to single-gene copy number variants was applied^36^.

### Copy number variation (CNV) analysis

Gain/loss of large DNA segments in the *BARD1* gene were predicted by the CNid method developed by Sistemas Genómicos based on a read depth (RD) approach. Briefly, this approach is based on the comparison of the tested sample with a pool of normal samples, under the hypothesis that depth of coverage in a genomic region is correlated with the copy number of the region^19^. Identified CNVs were confirmed by Multiplex Ligation-dependent Probe Amplification (MLPA). The assay was performed using the MLPA Probemix P489 BARD1 (MRC-Holland) according to manufacturer’s instructions. MLPA results were analyzed with Peak Scanner Software (Thermo Fisher Scientific). For MLPA analysis, each sample harbouring a candidate CNVs in the *BARD1* gene, was compared with five reference samples. Data were analysed and visualized using the Coffalyser.net software (MRC-Holland) (Supplementary Fig. 1). For the sample harbouring the single exon 9 deletion, sanger sequencing of the genomic region close to the ligation site of the half-probes (underlined in red) specific for exon 9, was performed to confirm the absence of single nucleotide polymorphisms that could have caused a decrease of the fluorescence signal (Supplemetary Fig. 1).

### Splicing studies

Synonymous variants were tested for its possible effect in splicing. Starting with RNA extracted from blood samples of the patients, a RT-PCR was performed and the cDNA obtained was amplified with specific primer pairs targeting the exons of interest (available upon request). PCR products were sequenced with the Sanger method using the Big Dye Terminator Cycle sequencing kit on an ABI 3730 Sequencer (Applied Biosystems). Results were visualized in FinchTV software.

## Supplementary Information

Supplementary Information 1.

Supplementary Information 2.

Supplementary Information 3.

## Data Availability

All variants reported in this study have been submitted to https://databases.lovd.nl/shared/genes/BARD1 with accession numbers 0000841043, 0000841044, 0000841045, 0000841046, 0000841047, 0000841048, 0000841050, 0000841051, 0000841052, 0000841053, 0000841054, 0000841055, 0000841058, 0000841059, 0000841060, 0000841061, 0000842164, 0000842165, 0000842166, 0000842167, 0000842168, 0000842169. All primer sequences used for Sanger validation of the variants are available upon request.
