# Supplementary material for: Apparent regional differences in the spectrum of BARD1 pathogenic variants in Spanish population and importance of copy number variants
Source: Sci Rep. 2022 May 20;12:8547. doi: 10.1038/s41598-022-12480-2 (PMC9122922; doi:10.1038/s41598-022-12480-2)
Supplement: Supplementary file 1 — Supplementary Information 1. [file 41598_2022_12480_MOESM1_ESM.docx]

**Apparent regional differences in the spectrum of *BARD1* pathogenic variants in Spanish population and importance of copy number variants.**

**Benito-Sánchez B^1^, Barroso A^1^*, Fernández V^1^*, Mercadillo F^1^, Núñez-Torres R^2^, Pita G^2^, Pombo L^3^, Morales-Chamorro R^4^, Cano-Cano JM^5^, Urioste M^1^, González-Neira A^2^, Osorio A^1,6**^.**

^1^Familial Cancer Clinical Unit, Human Cancer Genetics Programme, Spanish National Cancer Research Centre (CNIO), Madrid, 28029, Spain.

^2^Human Genotyping Unit (CEGEN), Human Cancer Genetics Programme, Spanish National Cancer Research Centre (CNIO), Madrid, 28029, Spain.

^3^Medical Oncology Section, Universitary Hospital Complex of Albacete, Albacete, Spain.

^4^Medical Oncology Section, Hospitalary Compex La Mancha Centro, Alcázar de San Juan, Ciudad Real, Spain.

^5^Medical Oncology Service, Universitary General Hospital of Ciudad Real, Ciudad Real, Spain.

^6^Spanish Network on Rare Diseases (CIBERER), Madrid, 28029, Spain.

*These authors contributed equally to this work.

**Corresponding Author: Ana Osorio, Familial Cancer Clinical Unit, Human Cancer Genetics Programme, Spanish National Cancer Research Centre (CNIO), C/Melchor Fernández Almagro 3, Madrid 29029, Spain. Phone: +34917328002, email: [aosorio@cnio.es](mailto:aosorio@cnio.es)

**A**
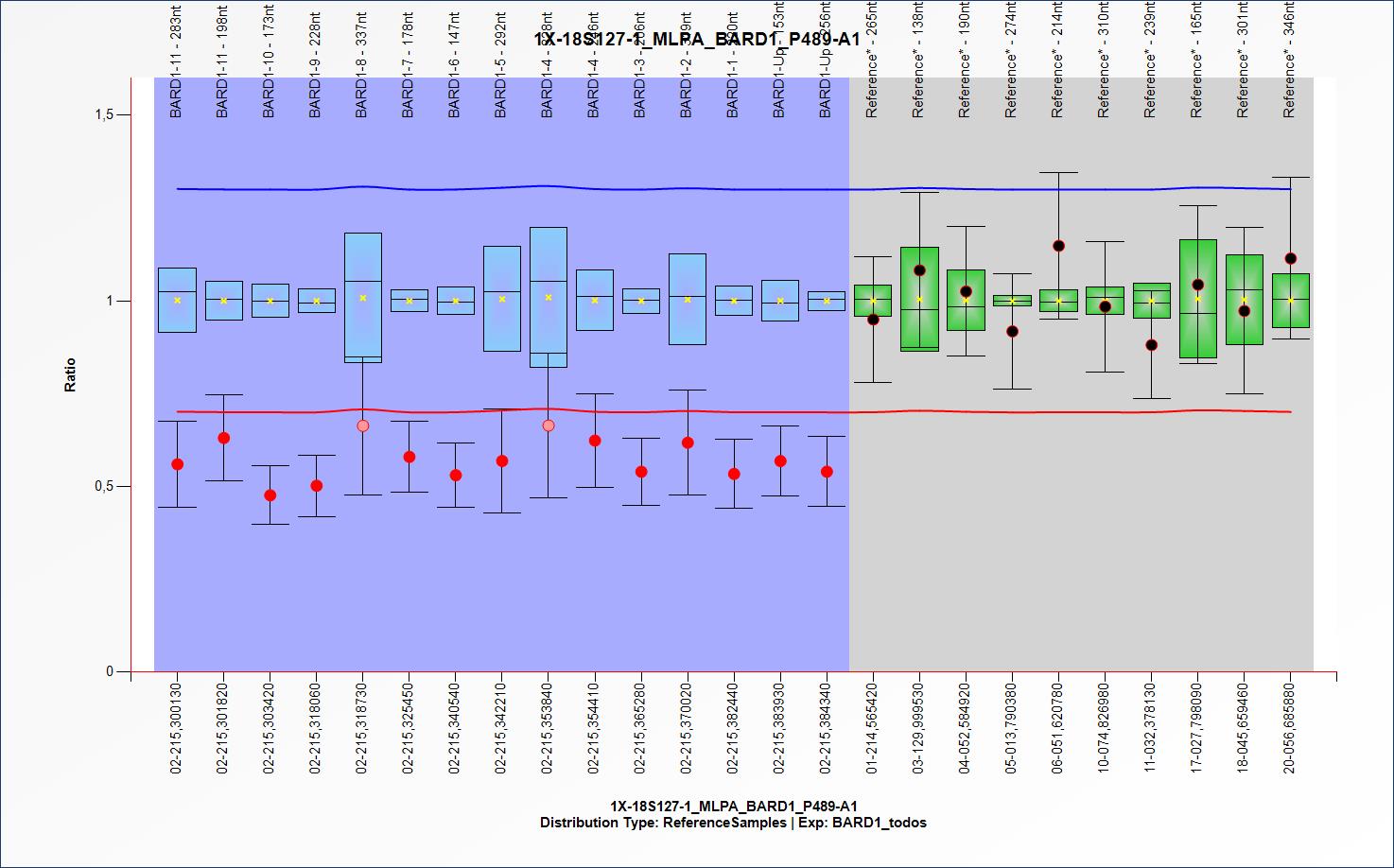


**B**


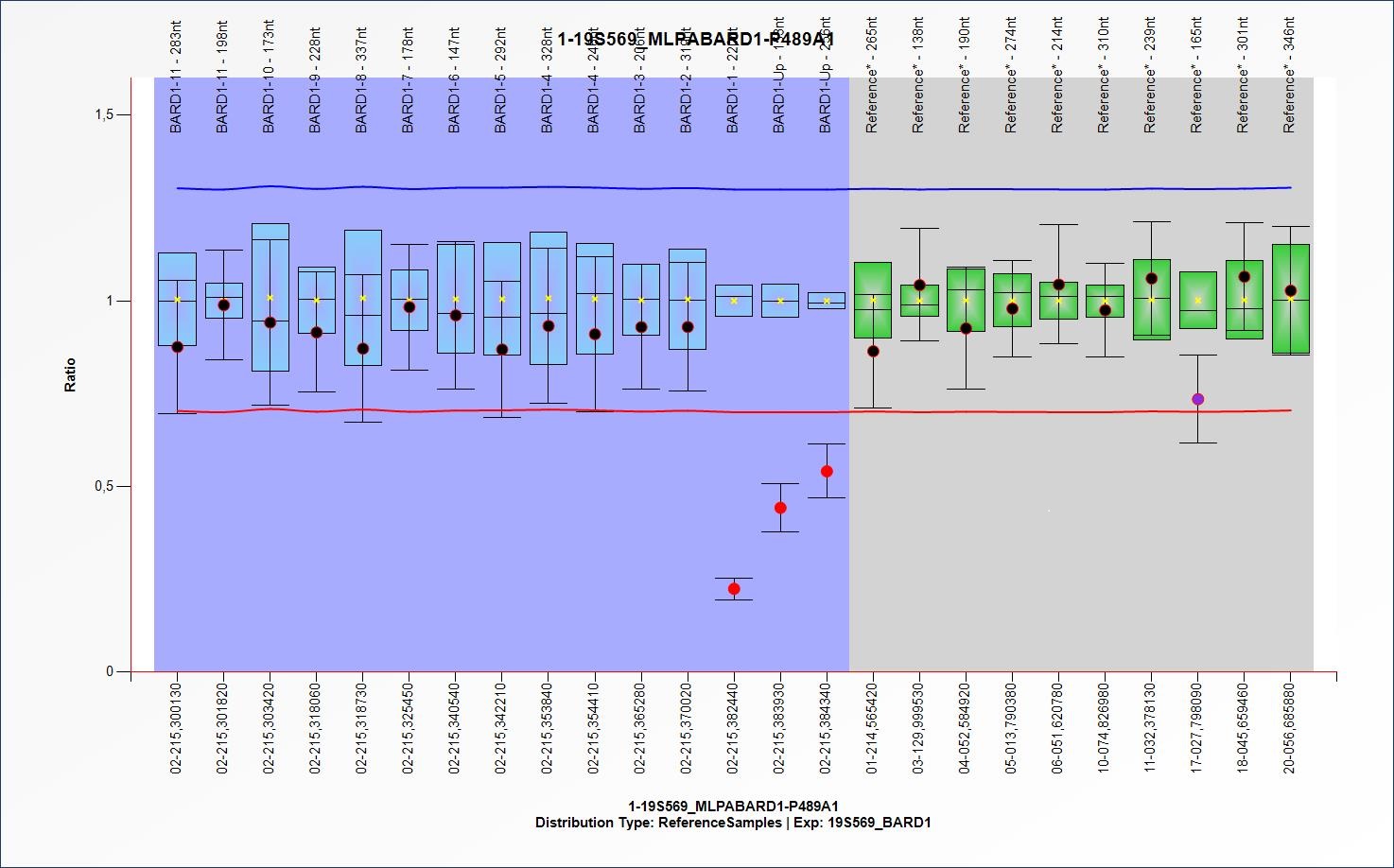


**C**
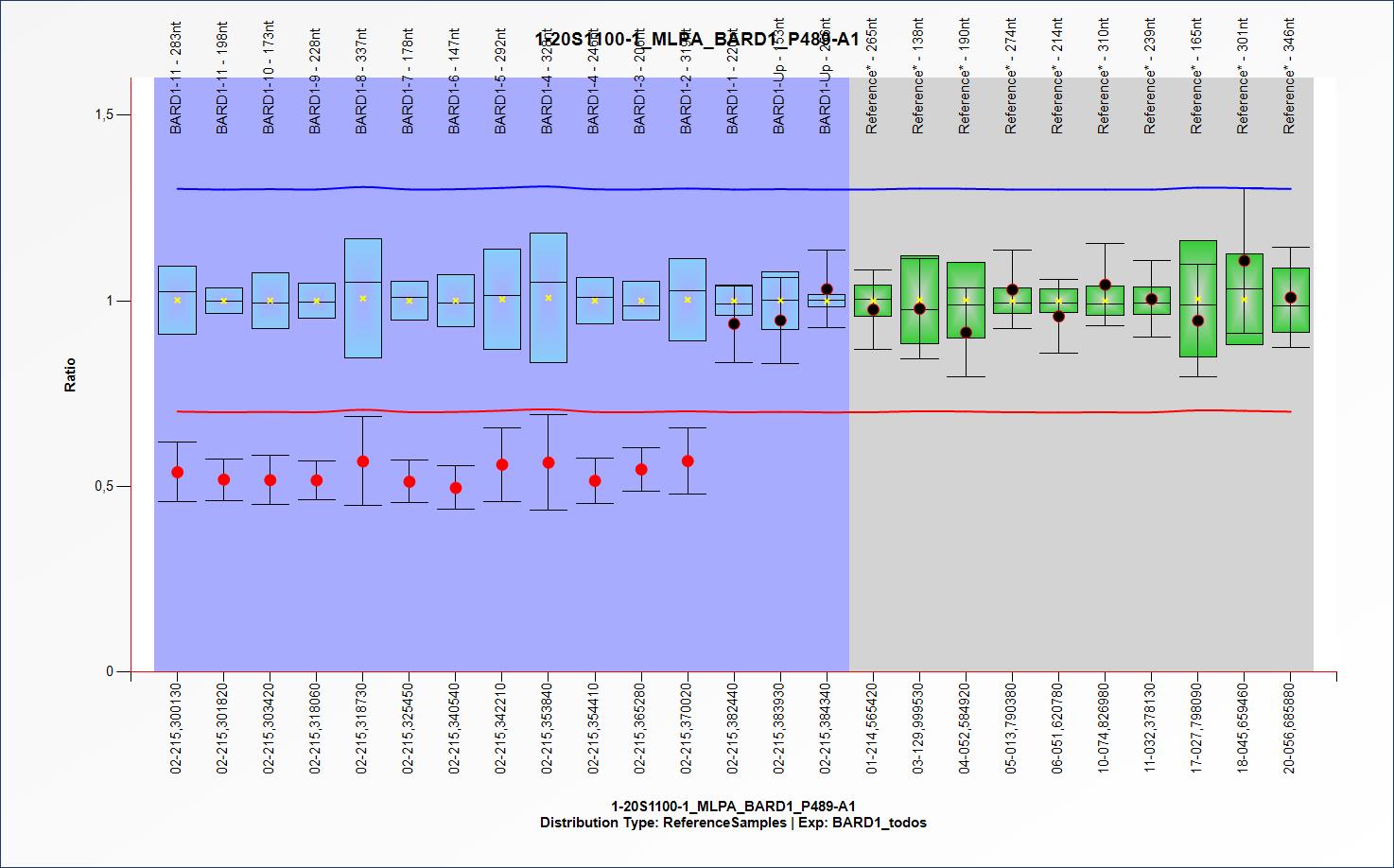


**D
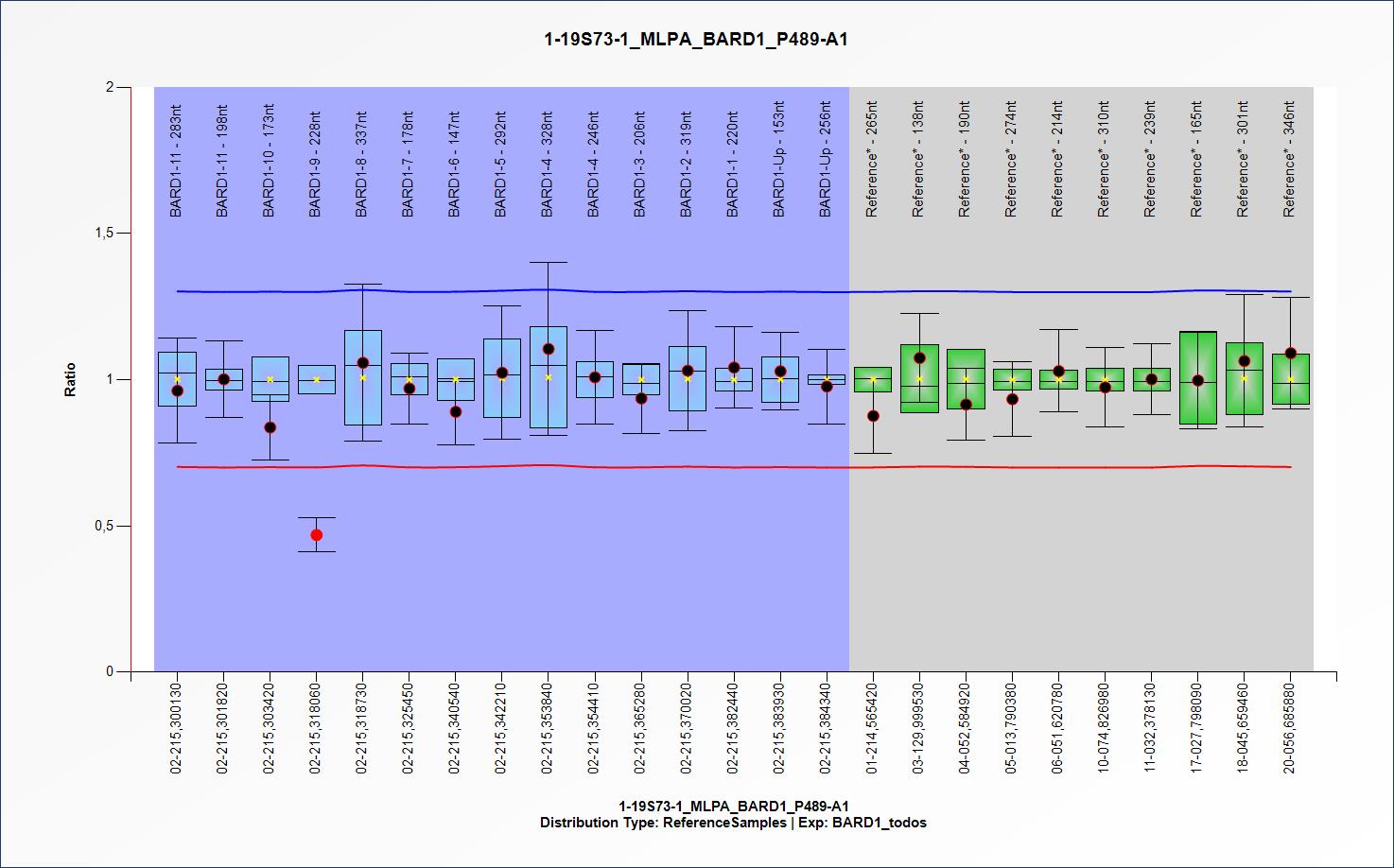
**

**E
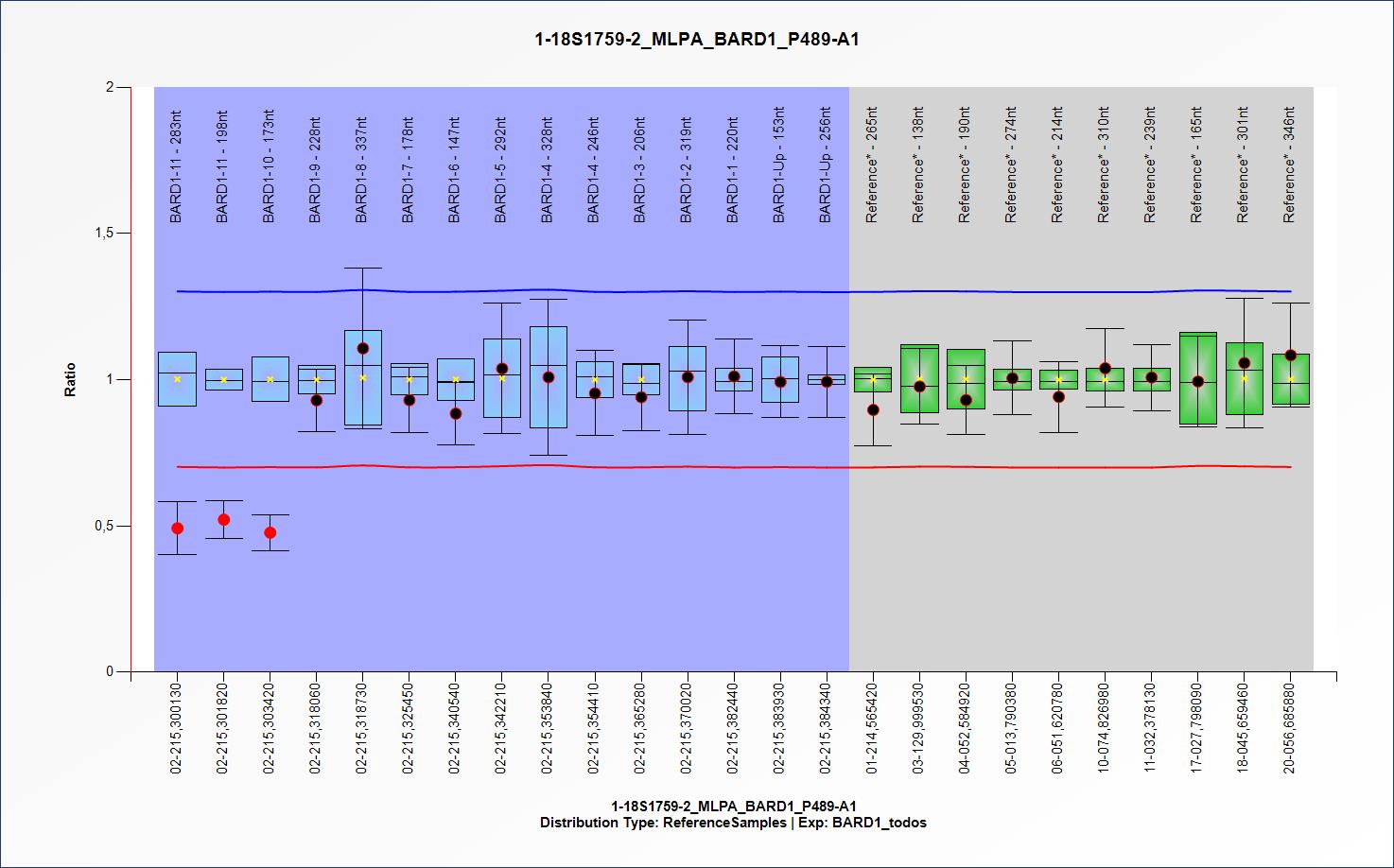
**

**F
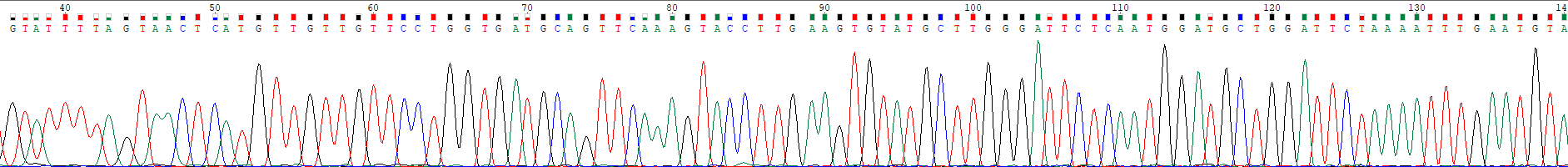
**

**Supplementary Figure S1**. Bar graphs obtained with the program Coffalyser (MRC- Holland) showing MLPA results from the five BC patients carrying large deletions in BARD1. Fifteen probes covering the 11 coding exons and 5’ UTR region of the BARD1 gene are represented by light blue vertical bars in the purple shaded half left area of the Bar graphs. Ten control probes from other genomic regions, are represented by green bars in the grey shaded right half area. Blue and red horizontal lines represent the threshold for the region to be considered as loss (ratio < 0.75) or gain (ratio >1.25) of one copy respectively. For each BARD1 or reference probe, the vertical bar represents the median and confidence interval of the fluorescence value of four control samples, the yellow star indicates the mean, and the circle represents the fluorescence and confidence interval obtained for the patient with a CNV. Red circles represent lost exons and black circles intact exons. (A) Deletion of the entire gene. (B) 5´UTR and Exon 1 deletion. (C) Exons 2-11 deletion. (D) Exon 9 deletion. (E) Exons 10-11 deletion. (F) Sanger sequencing of the sample harbouring the exon 9 single exon deletion in *BARD1* (D), showing the genomic region close to the ligation site of the half-probes (underlined in red) specific for exon 9, where the decrease fluorescence signal was detected.
