# Supplementary material for: Apparent regional differences in the spectrum of BARD1 pathogenic variants in Spanish population and importance of copy number variants
Source: Sci Rep. 2022 May 20;12:8547. doi: 10.1038/s41598-022-12480-2 (PMC9122922; doi:10.1038/s41598-022-12480-2)
Supplement: Supplementary file 2 — Supplementary Information 2. [file 41598_2022_12480_MOESM2_ESM.docx]

**Apparent regional differences in the spectrum of *BARD1* pathogenic variants in Spanish population and importance of copy number variants.**

**Benito-Sánchez B^1^, Barroso A^1^*, Fernández V^1^*, Mercadillo F^1^, Núñez-Torres R^2^, Pita G^2^, Pombo L^3^, Morales-Chamorro R^4^, Cano-Cano JM^5^, Urioste M^1^, González-Neira A^2^, Osorio A^1,6**^.**

^1^Familial Cancer Clinical Unit, Human Cancer Genetics Programme, Spanish National Cancer Research Centre (CNIO), Madrid, 28029, Spain.

^2^Human Genotyping Unit (CEGEN), Human Cancer Genetics Programme, Spanish National Cancer Research Centre (CNIO), Madrid, 28029, Spain.

^3^Medical Oncology Section, Universitary Hospital Complex of Albacete, Albacete, Spain.

^4^Medical Oncology Section, Hospitalary Compex La Mancha Centro, Alcázar de San Juan, Ciudad Real, Spain.

^5^Medical Oncology Service, Universitary General Hospital of Ciudad Real, Ciudad Real, Spain.

^6^Spanish Network on Rare Diseases (CIBERER), Madrid, 28029, Spain.

*These authors contributed equally to this work.

**Corresponding Author: Ana Osorio, Familial Cancer Clinical Unit, Human Cancer Genetics Programme, Spanish National Cancer Research Centre (CNIO), C/Melchor Fernández Almagro 3, Madrid 29029, Spain. Phone: +34917328002, email: [aosorio@cnio.es](mailto:aosorio@cnio.es)


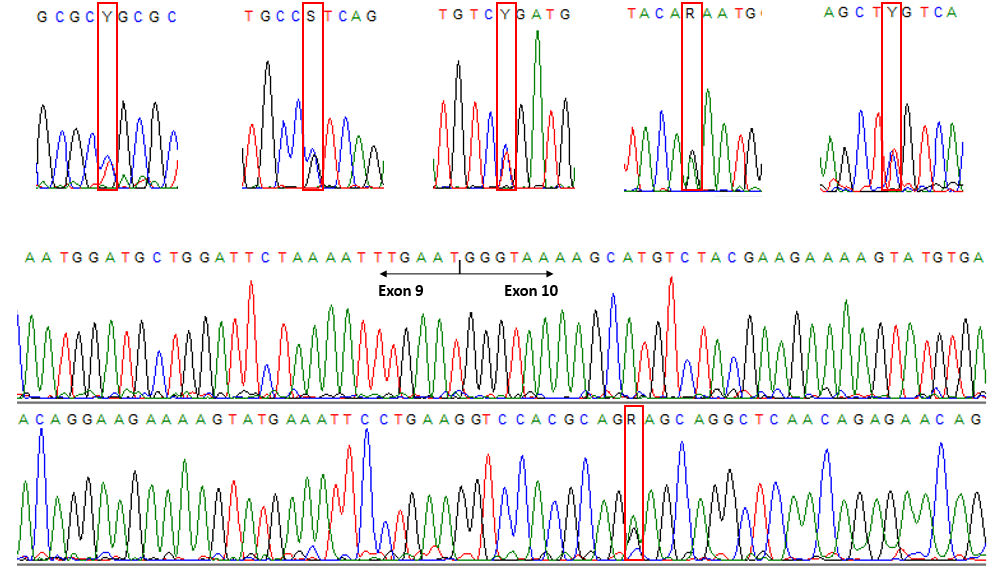
**A B C D E**


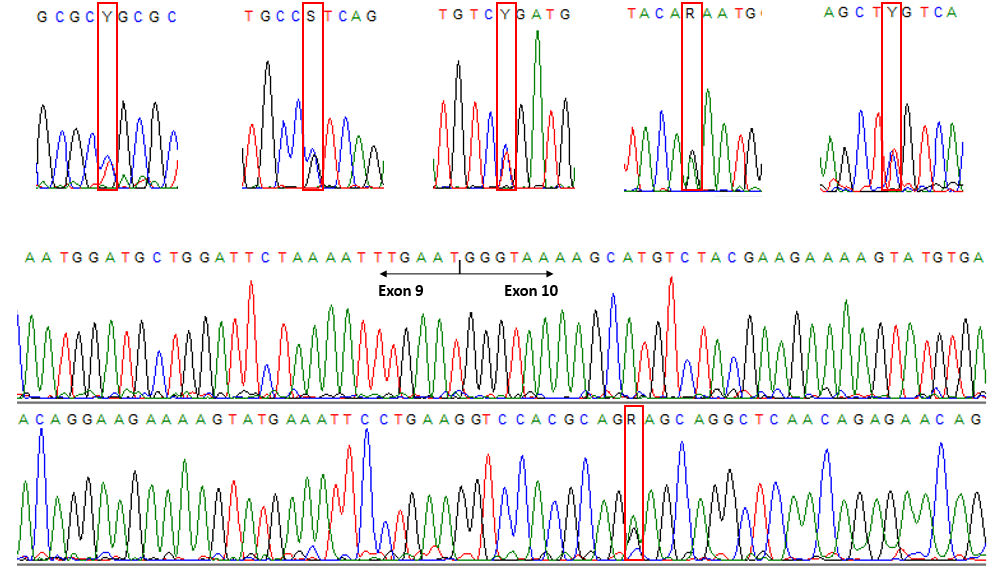
**F**

**Supplementary Figure S2**. Sanger sequencing chromatograms of the synonymous variants detected in our population. Red boxes indicate the nucleotide changes detected at the cDNA level, confirming the bialelic expression and no affectation of splicing. (A) c.117C>T. (B) c.1059C>G. (C) c.1152C>T. (D) c.1347A>G. (E) c.2082C>T. (F) c.1977A>G variant not showing exons 2-9 deletion (upper panel) and bialelic expression of the variant (lower panel) at the cDNA level.
