# Supplementary material for: Apparent regional differences in the spectrum of BARD1 pathogenic variants in Spanish population and importance of copy number variants
Source: Sci Rep. 2022 May 20;12:8547. doi: 10.1038/s41598-022-12480-2 (PMC9122922; doi:10.1038/s41598-022-12480-2)
Supplement: Supplementary file 3 — Supplementary Information 3. [file 41598_2022_12480_MOESM3_ESM.docx]

**Apparent regional differences in the spectrum of *BARD1* pathogenic variants in Spanish population and importance of copy number variants.**

**Benito-Sánchez B^1^, Barroso A^1^*, Fernández V^1^*, Mercadillo F^1^, Núñez-Torres R^2^, Pita G^2^, Pombo L^3^, Morales-Chamorro R^4^, Cano-Cano JM^5^, Urioste M^1^, González-Neira A^2^, Osorio A^1,6**^.**

^1^Familial Cancer Clinical Unit, Human Cancer Genetics Programme, Spanish National Cancer Research Centre (CNIO), Madrid, 28029, Spain.

^2^Human Genotyping Unit (CEGEN), Human Cancer Genetics Programme, Spanish National Cancer Research Centre (CNIO), Madrid, 28029, Spain.

^3^Medical Oncology Section, Universitary Hospital Complex of Albacete, Albacete, Spain.

^4^Medical Oncology Section, Hospitalary Compex La Mancha Centro, Alcázar de San Juan, Ciudad Real, Spain.

^5^Medical Oncology Service, Universitary General Hospital of Ciudad Real, Ciudad Real, Spain.

^6^Spanish Network on Rare Diseases (CIBERER), Madrid, 28029, Spain.

*These authors contributed equally to this work.

**Corresponding Author: Ana Osorio, Familial Cancer Clinical Unit, Human Cancer Genetics Programme, Spanish National Cancer Research Centre (CNIO), C/Melchor Fernández Almagro 3, Madrid 29029, Spain. Phone: +34917328002, email: [aosorio@cnio.es](mailto:aosorio@cnio.es)

**Supplementary Table 1.** Summary and final clinical classification of rare *BARD1* synonymous variants present in the 1946 patients analysed.

| **DNA level** | **Protein effect** | **Frequency in our cohort** | **gnomAD frequency^a^** | **ClinVar^b^** | **Franklin^c^** | **Final classification** |
| --- | --- | --- | --- | --- | --- | --- |
| c.117C>T | p.Ala39= | 0.0005138 | 0.00004586 | Likely benign | Likely benign | Benign |
| c.1059C>G | p.Pro353= | 0.0005138 | 0.00007164 | Benign/Likely benign​ | Likely benign | Benign |
| c.1152C>T | p.Ser384= | 0.0005138 | 0.0001274 | Unclassified:Likely benign/VUS | Likely benign | Benign |
| c.1347A>G | p.Gln449= | 0.0015416 | 0.0002122 | Unclassified:Benign/Likely benign/VUS | Likely benign | Benign |
| c.1977A>G | p.Arg659= | 0.0056526 | 0.002037 | Unclassified:Benign/Likely benign/VUS​ | Likely benign | Benign |
| c.2082C>T | p.Leu694= | 0.0005138 | 0.0001769 | Unclassified:Benign/Likely benign/VUS | Likely benign | Benign |

*VUS=* variant of uncertain significance.

^a^ Total allele frequency for all populations described in gnomAD (https://gnomad.broadinstitute.org/).

^b^ Aggregate clinical significance from all records at ClinVar (https://www.ncbi.nlm.nih.gov/clinvar/).

^c^ Clinical classification using ACMG criteria generated by the advanced artificial intelligence Franklin by Genoox (https://franklin.genoox.com/clinical-db/home).
